# Real Time Monitoring of a UV Light-Assisted Biofunctionalization Protocol Using a Nanophotonic Biosensor

**DOI:** 10.3390/bios9010006

**Published:** 2018-12-30

**Authors:** Jad Sabek, Luis Torrijos-Morán, Amadeu Griol, Zeneida Díaz Betancor, María-José Bañuls Polo, Ángel Maquieira, Jaime García-Rupérez

**Affiliations:** 1 Nanophotonics Technology Center, Universitat Politècnica de València, Camí de Vera s/n, 46022 Valencia, Spain; Luitorm2@ntc.upv.es (L.T.-M.); agriol@upvnet.upv.es (A.G.); 2 Departamento de Química, Instituto Interuniversitario de Investigación de Reconocimiento Molecular y Desarrollo Tecnológico IDM, Universitat Politècnica de València, Camí de Vera s/n, 46022 Valencia, Spain; zediabe@upvnet.upv.es (Z.D.B.); mbpolo@upvnet.upv.es (M.-J.B.P.); amaquieira@qim.upv.es (Á.M.)

**Keywords:** biofunctionalization, UV light photocatalysis, half antibodies, silicon on insulator, nanophotonic sensor

## Abstract

A protocol for the covalent biofunctionalization of silicon-based biosensors using a UV light-induced thiol–ene coupling (TEC) reaction has been developed. This biofunctionalization approach has been used to immobilize half antibodies (hIgG), which have been obtained by means of a tris(2-carboxyethyl)phosphine (TCEP) reduction at the hinge region, to the surface of a vinyl-activated silicon-on-insulator (SOI) nanophotonic sensing chip. The response of the sensing structures within the nanophotonic chip was monitored in real time during the biofunctionalization process, which has allowed us to confirm that the bioconjugation of the thiol-terminated bioreceptors onto the vinyl-activated sensing surface is only initiated upon UV light photocatalysis.

## 1. Introduction

In recent years, great interest has been shown in the development of high-performance lab-on-a-chip (LOC) biosensing devices that are able to replace conventional methodologies currently used, such as PCR or ELISA, which are expensive, bulky, time-consuming, and lab-centralized [1,2]. The combination of microfluidics and nanoscale transduction elements based on different mechanisms, such as optical, electrical, or mechanical [3,4,5], allows to perform the required analyses with high sensitivity, a high degree of miniaturization, a high multiplexing level, shorter time to results and requiring very low volumes of sample. However, these transduction elements are typically not able to provide a specific response towards a certain analyte/substance, so a so-called biofunctionalization of the sensing structures is require to immobilize specific bioreceptors towards that analyte/substance [6]. Among the different procedures for the biofunctionalization of biosensing structures, covalent strategies for the immobilization of bioreceptors provide several advantages in terms of nonspecific interactions restriction, robustness in the attachment, and thickness reduction of the recognition layer, compared to other strategies based on physical adsorption [7].

Here, we report our work towards the development of a UV light-induced biofunctionalization protocol for the covalent immobilization of specific bioreceptors over silicon-based biosensing structures. The use of a UV light-induced biofunctionalization method allows a high resolution and spatially selective immobilization only in those positions being irradiated with UV light. A biofunctionalization protocol based on the thiol–ene coupling (TEC) reaction [8,9], which occurs between thiol and vinyl groups upon UV light excitation, has been used to immobilize thiol-terminated bioreceptors over vinyl-activated silicon-on-insulator (SOI) photonic bandgap (PBG) sensing structures. Half-antibodies (hIgG) have been used as bioreceptors for the immobilization as they provide free thiol moieties available after the cleavage of their disulfide bridges. The biofunctionalization of the silicon-based sensing structures within the photonic chip has been carried out online, while their response was monitored in real time, which has allowed us to experimentally confirm the photocatalytic reaction required to immobilize the bioreceptors using the TEC approach.

## 2. Materials and Methods

### 2.1. Silanization of the Silicon Surface

**Figure 1:**
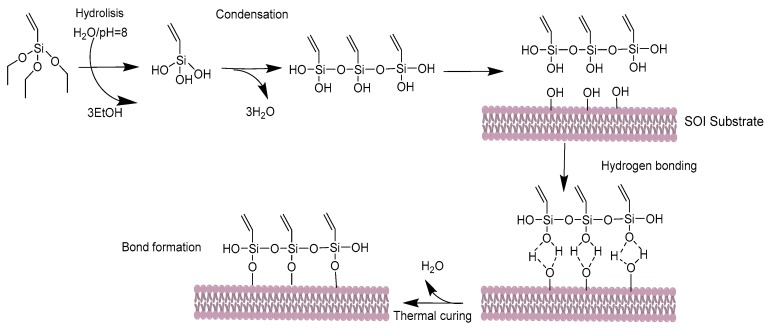
Steps of the silanization process used to create a vinyl-terminated monolayer on the surface of the silicon-on-insulator (SOI) sensing structures.

In order to immobilize the thiol-terminated bioreceptors onto the silicon-based sensors using the UV light-induced TEC reaction [8,9], the silicon-based photonic chip is first silanized using triethoxyvinyl–silane (TEVS) to obtain a vinyl-terminated monolayer on its surface. The steps of the silanization process used in this work are depicted in Figure 1. Typically, silanization processes are carried out using an organic anhydrous solvent as the carrier for the organosilane, as previously done for the creation of fluorescence microarrays [10,11]. However, the use of organic solvents can lead to problems such as vertical polymerization, which produces a thicker organosilane layer that increases the distance between the sensor surface and the target and can reduce sensitivity, as well as the generation of organic waste. Considering these problems, we have performed the silanization process using water as the carrier for the organosilane, as its use has also been demonstrated as adequate for the biofunctionalization of silicon-based biosensors [12]. Although the solubility of TEVS in water is not complete, the exchange of its triethoxy groups with OH groups from water makes it stable. In order to have an optimal triethoxy ↔ OH exchange ratio and to finally obtain a compact vinyl monolayer on the surface, the pH of the solution is adjusted to 8 using KOH or NaOH. Therefore, the final silanization process consisted of immersing the SOI photonic chip in 1% TEVS in Milli-Q water (pH adjusted to 8 by adding 100 µL of 1 M KOH in Milli-Q water solution) for 1 h and finally curing it at 110 °C for 1 h for condensation and water excess evaporation. Note that before performing the silanization process, the SOI photonic chip was cleaned in a piranha solution (H_2_SO_4_/H_2_O_2_: 1/3) for 20 min and then activated using O_2_ plasma for 10 min.

### 2.2. UV Light-Assisted Immobilization of Half-Antibodies

**Figure 2:**
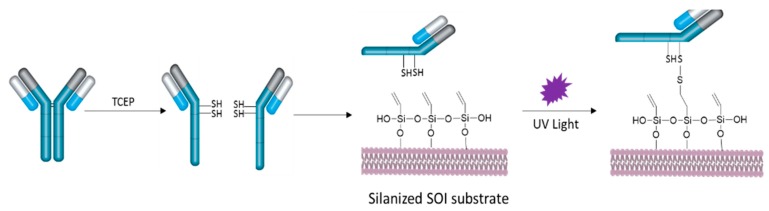
Schematic representation of the process used for the UV light-assisted covalent immobilization of the half anti-bovine serum albumin (haBSA) on the SOI sensors surface.

The thiol-terminated bioreceptors used in this work are hIgG specific to bovine serum albumin (BSA). These half anti-BSA antibodies (haBSA) were obtained using a tris(2-carboxyethyl)phosphine (TCEP) reduction process consisting of the incubation (for 90 min at 37 °C) of the anti-BSA in acetate buffer (0.15 M sodium acetate, 0.01 M EDTA, 0.1 M sodium chloride, pH = 4.5) at 4 mg/mL concentration in the presence of 25 mM TCEP. The corresponding haBSA were purified by employing a 50 kDa centrifugal filter unit and the concentrations of the solutions were determined by employing a NanoDrop spectrophotometer. After this cleavage process, thiol groups from the disulphide bridges became available on the resulting haBSA for their immobilization over the vinyl-terminated surface by means of UV light (254 nm) photocatalysis, as schematically depicted in Figure 2. Further details about this TCEP reduction process can be found in Reference [11]. Note that the use of immobilized hIgG with the biofunctionalization approach described in this work can also provide several significant benefits as a higher surface coverage density, a lower thickness of the biorecognition layer, and a proper orientation of the antibodies binding sites.

### 2.3. SOI Photonic Sensing Chip

**Figure 3:**
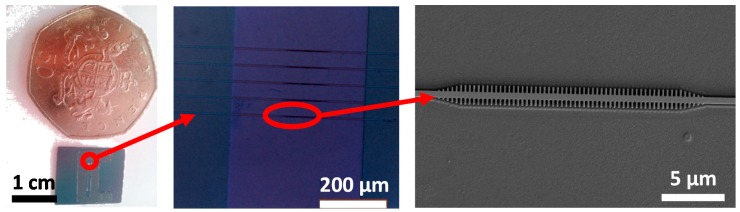
(**Left**) Image of the photonic chip fabricated in SOI technology. The circle depicts the position of one of the photonic bandgap (PBG) sensor groups. (**Center**) Microscope image of a PBG sensor group. The fifth structure on the top of the group corresponds to a reference waveguide. The channel opened on the SiO_2_ upper cladding can also be observed. The circle depicts an individual PBG sensing structure. (**Right**) Scanning electron microscope (SEM) image of a fabricated PBG sensing structure.

**Figure 4:**
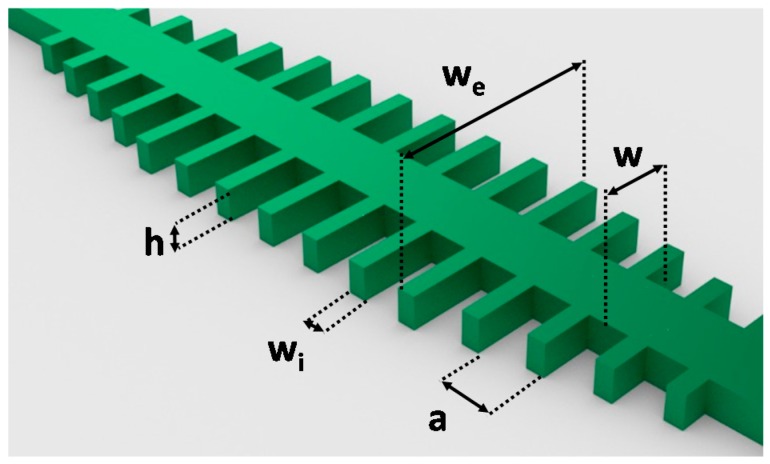
Schematic representation of the PBG sensing structure and its structural parameters.

Figure 3 shows several pictures of the SOI photonic chip used to monitor in real time the UV light-assisted immobilization of the haBSA. It contains several PBG structures that are used as sensing elements. In these structures, the introduction of a periodic modulation in the refractive index of the photonic structure gives rise to the appearance of a rejected spectral band, the so-called PBG [13], whose position will depend on the refractive index of the surrounding medium with a high sensitivity. The SOI photonic sensing chip has been created in our clean room facilities using e-beam lithography to expose the chip layout on a layer of hydrogen silsesquioxane (HSQ) resist and then transferring that layout to the top silicon layer of the SOI chip using inductively coupled plasma etching. The SOI photonic chip contains 4 groups of 4 PBG sensing structures whose structural parameters are (see Figure 4) height h = 220 nm, waveguide width w = 450 nm, period a = 380 nm, transversal elements length w_e_ = 1500 nm, and transversal elements widths ranging from w_i_ = 80 nm to 140 nm (w_i_ = 80, 100, 120, and 140 nm for each of the PBG structures within each group). These structural parameters provide PBG edges located in the 1550 nm wavelength range, where our experimental characterization equipment operates. The separation between the sensor groups is 1.5 mm in the transversal direction of the photonic chip. The chip is accessed at the input and the output via 70 nm-deep shallow etch 1D grating couplers. Finally, the chip is covered with a 400 nm-thick SiO_2_ upper cladding and a 400 µm-wide channel is opened on it using UV lithography in order to have access to the PBG sensing structures.

### 2.4. Experimental Platform

**Figure 5:**
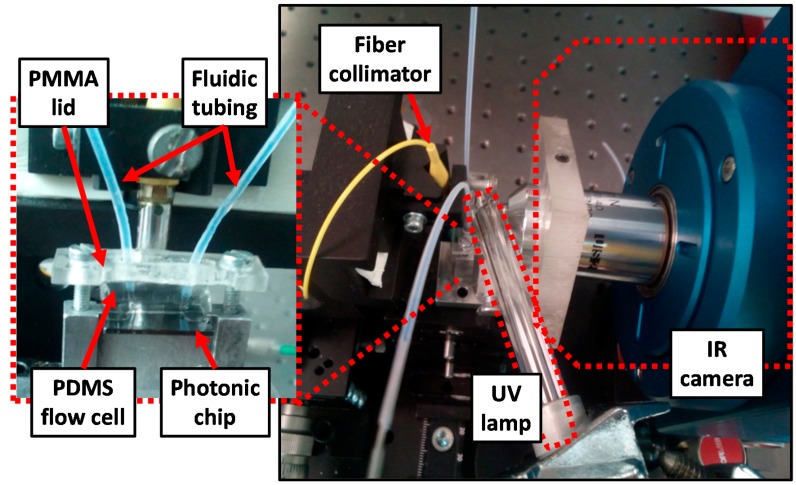
Experimental setup employed for the interrogation of the photonic chip containing the PBG sensing structures. The zoom image depicts the assembled photonic + fluidic chip. PMMA: Polymethyl methacrylate, PDMS: Polydimethylsiloxane, IR: Infrared.

Figure 5 depicts the experimental platform used to monitor in real time the response of the photonic sensing structures during the implementation of the biofunctionalization process. Once the SOI photonic chip is silanized in order to obtain a vinyl-terminated monolayer on its surface, it is assembled with a polydimethylsiloxane (PDMS) microfluidic flow cell having a channel of size 400 µm × 50 µm × 6 mm (width × height × length) which is accessed via 2 polytetrafluoroethylene (PTFE) tubes. The assembled photonic + fluidic chip is placed on the sample holder and fixed using a polymethyl methacrylate (PMMA) lid. PDMS and PMMA have been used for the realization of the flow cell and the lid, respectively, as they are transparent to UV light and will allow to irradiate the photonic chip during the characterization of the photocatalyzed biofunctionalization process. In order to characterize the photonic chip, light from a continuous sweep tunable laser is coupled to the input grating couplers using a fiber collimator. Light coming out from the output grating couplers is measured using an infrared (IR) camera synchronized with the tunable laser in order to obtain the spectra of all the PBG sensing structures within the chip simultaneously [14]. The target solutions are flowed using a syringe pump working in withdraw mode and set to a constant flow rate of 10 μl/min to ensure laminar flow [15].

## 3. Results and Discussion

**Figure 6:**
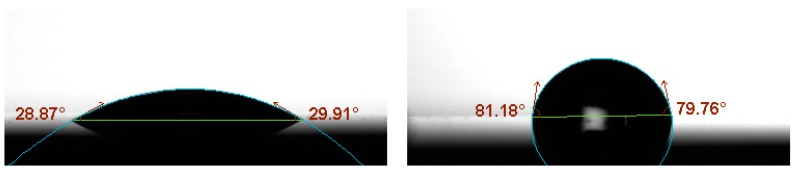
Water contact angle (WCA) test for (**left**) the bare SOI chip and (**right**) the SOI chip after triethoxyvinyl–silane (TEVS) silanization.

**Figure 7:**
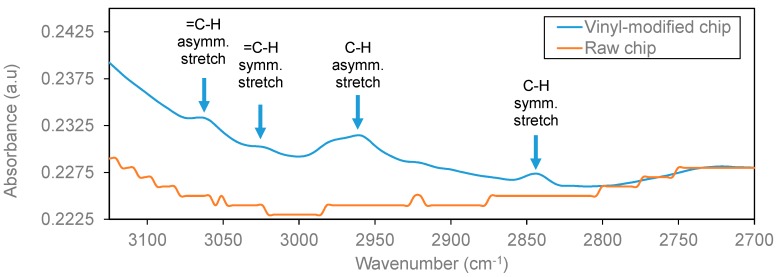
Infrared reflection absorption spectroscopy (IRRAS) characterization results of the SOI chip before and after the TEVS silanization. Bands at 3062 and 3020 cm^−1^ indicate the presence of vinyl groups.

**Figure 8:**
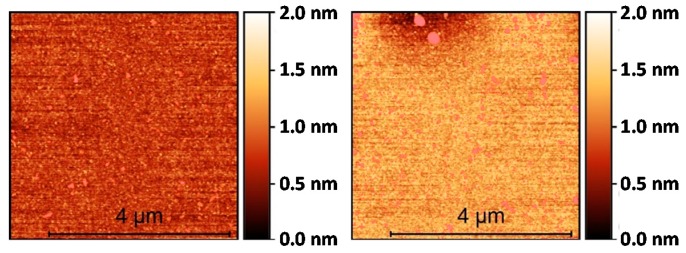
Atomic force microscope (AFM) topography characterization of (**left**) the bare SOI surface and (**right**) the SOI surface after TEVS silanization. A very low roughness (in the sub-nm range) is measured for both surfaces.

In order to characterize the initial silanization step, several characterization techniques were employed before and after performing it. First, the water contact angle (WCA) test indicates a significant increase of the hydrophobicity of the surface after performing the TEVS silanization (from ~30° to ~80°, as shown in Figure 6), which confirms the coverage of the SOI surface with the organosilane. Then, IRRAS (infrared reflection absorption spectroscopy) was employed to characterize the surface composition (see Figure 7). The spectral bands appearing at 3062 cm^−1^ and 3020 cm^−1^ correspond to the =C-H asymmetric and symmetric stretching vibrations for the vinyl groups of the TEVS organosilane [16], which confirms the presence on the SOI surface of those vinyl groups required to perform the proposed light-assisted immobilization of thiol-terminated probes. Finally, the topography of the surface before and after TEVS functionalization was characterized by means of AFM (atomic force microscope) measurements. Figure 8 demonstrates that a very low roughness is measured after performing the silanization process, which indicates a high homogeneity and compactness of the deposited TEVS layer. Therefore, all the characterization techniques employed confirm the adequate creation of the TEVS layer on the surface of the SOI chip.

**Figure 9:**
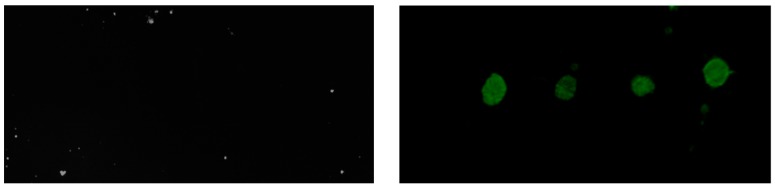
Fluorescence microarray measurements for two TEVS silanized chips where fluorophore labelled haBSA (haBSA*) has been deposited. The chip in the left has not been irradiated with UV light, so the lack of fluorescence indicates that the haBSA* has not been attached to the surface. The chip in the right has been irradiated with UV light after the deposition of the haBSA*, so the presence of fluorescence indicates the successful immobilization due to the thiol–ene coupling (TEC) reaction.

Once the proper silanization of the chip surface was confirmed, we performed fluorescence microarray tests on planar SOI surfaces to verify the proposed light-assisted TEC immobilization protocol. To this aim, fluorophore labelled haBSA (haBSA*) spots were deposited onto two different TEVS silanized SOI chips. One of those chips was irradiated with UV light (at 254 nm with a power of 6 mW/cm^2^) to induce the reaction between the vinyl groups on the surface and the thiol groups from the haBSA*, while the other chip was kept without UV light irradiation. Finally, both chips were thoroughly washed with phosphate buffered saline with Tween 20 (PBS-T) and water and dried, and the fluorescence was checked. Figure 9 shows the fluorescence results for both chips, where we can clearly see that fluorescence is only observed for the chip being irradiated with UV light, thus confirming the UV-dependent nature of the biofunctionalization process, as it was previously observed when using toluene as the carrier for the silanization process [11].

**Figure 10:**
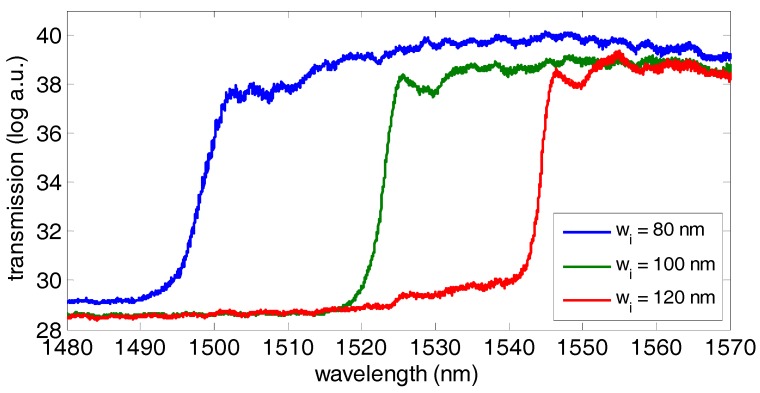
Initial spectrum in phosphate buffered saline (PBS) 1× for the PBG sensing structures within one sensor group (transmission units are given by the analog-to-digital converter (ADC) values provided by the IR camera). Note that the spectrum for the PBG sensing structure with w_i_ = 140 nm is not depicted because its PBG edge is above the measurement wavelength range.

The next step is transferring the assay to the PBG sensing structures in order to monitor the light-assisted biofunctionalization process in real time. Figure 10 shows the initial spectra in phosphate buffered saline (PBS) 1× of the photonic sensing structures from one sensor group having its PBG located within the measurement range (the PBG edge for the w_i_ = 140 nm structure was not observed because it fell outside our measurement wavelength range). We can see that the increase of the width of the transversal elements w_i_ produces a shift of the PBG edge towards longer wavelengths. In order to perform the sensing, the position of the lobe appearing at the PBG edge will be tracked to monitor the spectral shift when the biofunctionalization events take place on the surface of the sensors.

**Figure 11:**
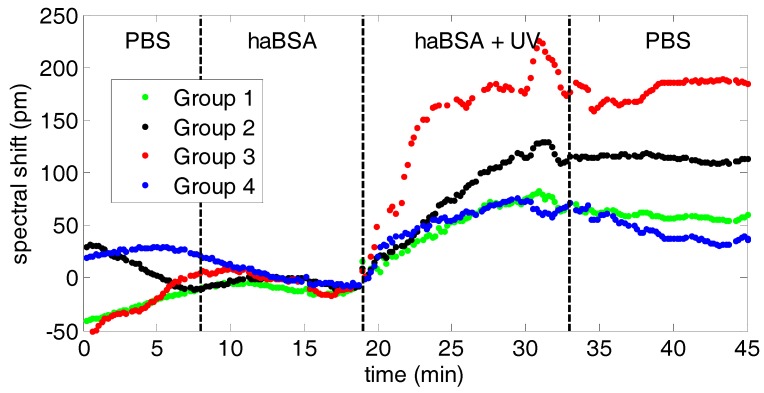
Temporal evolution of the PBG spectral shift during the real time monitoring of the UV light-assisted immobilization of the thiol-terminated haBSA over the vinyl-terminated SOI surface. haBSA are injected at minute 8 and the UV light source is switched on at minute 19.

Figure 11 shows the real time monitoring results obtained for the UV light-assisted immobilization of half-antibodies over the photonic chip. The sensing response from those PBG sensing structures having a width of the transversal elements of w_i_ = 120 nm for the 4 sensor groups is depicted. Initially, PBS 1x is flowed over the vinyl-terminated photonic chip to obtain the initial baseline. Then, the solution containing the thiol-terminated haBSA (20 µg/mL in PBS 1×) is flowed. As can be observed in Figure 11, no photonic sensing response is obtained at that moment for any of the PBG sensing structures even though both the surface vinyl groups and the thiol moieties of the haBSA are present. It is not until the photonic chip is irradiated with UV light (at 254 nm with a power of 6 mW/cm^2^) that the vinyl-thiol reaction is photocatalyzed and the haBSA are immobilized on the sensor surface, which is translated into a shift of the PBG position. Finally, PBS 1× buffer is flowed again to determine the net spectral shift and to remove any excess of haBSA.

We can observe that the sensing response is different for each PBG sensing structure belonging to a different sensors group, being higher for those sensor groups placed in a central location of the chip (groups 2 and 3). This is related with the fact that a perfectly homogeneous illumination of the whole photonic chip surface has not been possible due to the presence of the PDMS flow cell, the PMMA lid, and the PTFE tubing on top of the photonic chip, as well as by the limited space available to place the UV lamp in the experimental platform (see Figure 5). In this context, a better illumination of the central PBG sensors (groups 2 and 3) is produced, which is translated into a higher haBSA immobilization efficiency for those sensors, which is reflected into a higher spectral shift.

## 4. Conclusions

In this work, we have implemented a UV light-assisted biofunctionalization protocol for the immobilization of thiol-terminated bioreceptors onto vinyl-terminated silicon-based sensors. The fact that UV light is required to induce the immobilization allows that only those specific positions where the sensing structures are placed are biofunctionalized, which opens the door to obtaining a huger density of nanoscale biosensing structures being biofunctionalized with different bioreceptors for a massive scale multiplexing level. We have been able to monitor this photocatalytic immobilization in real time using an integrated nanophotonic sensing chip, showing that vinyl groups in the surface and thiol groups from the bioreceptors do not react until UV light is present. This result shows the feasibility of using nanophotonic sensors as a tool to study the mechanisms of photo-induced reactions in real time. Additionally, this is, to our knowledge, the first time that photonic sensing structures are biofunctionalized with half antibodies, which can also mean an advantage in terms of higher surface coverage density, lower thickness of the recognition layer, and proper orientation of the antibodies’ binding sites.
